# Expression Cloning of Recombinant *Escherichia coli *lacZ Genes Encoding Cytoplasmic and Nuclear β-galactosidase Variants

**Published:** 2011

**Authors:** Homayoun Naderian, Zahra Rezvani, Mohammad Ali Atlasi, Hossein Nikzad, AF de Vries Antoine

**Affiliations:** 1 * Anatomical Sciences Research Center, Kashan University of Medical Sciences, Kashan, Iran*; 2 * Department of Molecular Cell Biology, Leiden University Medical Center, Leiden, the Netherlands*

**Keywords:** Expression cloning, HeLa cells, lacZ gene, pcDNA3.1/myc-His C, Polyethylenimine, Transfection

## Abstract

**Objective(s):**

Nonviral vector can be an attractive alternative to gene delivery in experimental study. In spite of some advantages in comparison with the viral vectors, there are still some limitations for efficiency of gene delivery in nonviral vectors. To determine the effective expression, the recombinant *Escherichia coli *lacZ genes were cloned into the different variants of pcDNA3.1 and then the mammalian cells were transfected.

**Methods and Materials:**

The coding sequences of cytoplasmic and nuclear variants of lacZ gene were inserted downstream of the human cytomegalovirus immediate-early gene promoter of plasmid pcDNA3.1/myc-His C. The new cytoplasmic and nuclear constricts of *E. coli *β-galactosidase-coding sequences were introduced into HeLa cells with the aid of linear polyethylenimine and at 2 days post-transfection the cells were stained using 5-bromo-4-chloro-3-indolyl-β-D-galactopyranoside (X-gal).

**Results:**

Restriction enzyme analyses revealed the proper insertion of *E. coli *β-galactosidase-coding sequences into the multiple cloning site of pcDNA3.1/myc-His C. The functionality of the resulting constructs designated pcDNA3.1-cyt.lacZ and pcDNA3.1-nls.lacZ(+) was confirmed by X-gal staining of HeLa cells transfected with these recombinant plasmids. While pcDNA3.1-cyt.lacZ directed the synthesis of cytoplasmically located β-galactosidase molecules, the β-galactosidase protein encoded by pcDNA3.1-nls.lacZ(+) was predominantly detected in the cell nucleus.

**Conclusion:**

The expression of cytoplasmic and nuclear variant of LacZ gene confirmed the ability of pcDNA3.1 as versatility nonviral vector for the experimental gene delivery study in mammalian cells

## Introduction

Both viral and nonviral vectors are currently being developed into gene delivery vehicles for therapeutic applications (1). Although viral vectors have great potential as gene delivery vehicles, they also possess some disadvantages, including the risk of insertional mutagenesis when using non-specifically integrating vector systems. In addition, the size of therapeutic nucleic acid molecules that can be accommodated by viral vectors is limited by their restricted packaging capacity and viral vectors may induce potentially harmful innate and adaptive immune responses (2). They are also undesirable for DNA vaccine and cause the immune reaction against viral vector (3). Several of these problems may be overcome by using nonviral vectors, which are relatively easy to produce and have less safety concerns than viral vectors (4). When pursuing gene transfer delivery system on the basis of nonviral vectors, it is important to select an expression plasmid that directs high-level transgene expression in the target cells. pcDNA3 and its derivatives are among the most frequently used commercially available mammalian expression plasmids mainly because of their versatility, e.g. they have been used for vaccine purposes (5,6) and cancer gene therapy (7-9). 

A fast manner to test the utility of gene transfer vectors is through the use of reporter genes. One of the most popular reporter genes is the lacZ gene of *Escherichia coli* (10), which codes for the enzyme β-galactosidase (βgal, EC 3.2.1.23) (11). LacZ expression can be easily detected *in vivo *and *in vitro*, in fixed and living cells using qualitative (e.g. cyto/histochemical and immunological) as well as quantitative (e.g. colorimetric, chemiluminescent) assays (12). Moreover, the lacZ gene has been successfully transferred to many eukaryotic cells (13,14), applied as a reporter gene in the comparative analysis of gene repair strategies (15) and used for *in vivo* imaging of (trans) gene expression (16). 

The aim of this study was to investigate whether the mammalian expression plasmid pcDNA3.1/myc-His C could be used to direct the synthesis in human cells of recombinant *E. coli *β-galactosidase proteins with (nls.βgal) and without (cyt.βgal) the nuclear localization signal (nls) of simian virus 40 (SV40). We reported the generation and functional testing in mammalian transfection experiments of two different lacZ expression plasmids pcDNA3.1-cyt.lacZ and pcDNA3.1-nls.lacZ(+) that might be highly useful in the development of nonviral vectors and intracellular traffic agents for experimental gene delivery study. 

## Materials and Methods

***Molecular cloning procedures***

**Figure 1.**
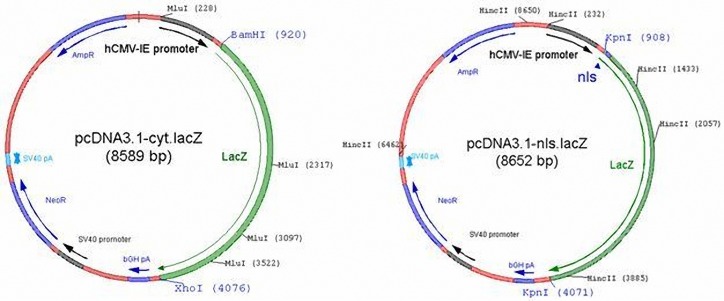
Map of plasmids pcDNA3.1-cyt.lacZ and pcDNA3.1-nls.lacZ(+). The RE recognition sites used for the insertion of the cyt-βgal- and nls-βgal-coding sequences are indicated in blue while the RE recognition sites used for screening are shown in black. AmpR = β-lactamase/ampicillin resistance gene; bGH pA = bovine growth hormone gene polyadenylation signal; NeoR = aphA1/neomycin resistance gene.

To generate the plasmid pcDNA3.1-cyt.lacZ (Figure 1), the cyt.βgal-coding sequence of pCMV SPORT-βgal (Invitrogen) was amplified by polymerase chain reaction (PCR) using primers cyt.βgal.F #720 (5’ CTAGGATCCGTCACC**ATG**TCGTTTACTTTGACC 3’; lacZ initiation codon shown in boldface) and cyt.βgal.R #721 (5’ GAACTCGAG**TTA**TTTTTGACACCAGACCAACTGG 3’; complement of lacZ termination codon shown in boldface) containing extragenic BamHI and XhoI recognition sites (underlined), respectively. The PCR was carried out with the aid of the High Fidelity PCR Enzyme Mix (Fermentas) according to the instructions of the supplier. After incubating pCMV SPORT-βgal (1 ng) for 2 min at 94 °C to separate the DNA strands, the template DNA was subjected to 30 rounds of amplification consisting of 30 sec at 94 °C (denaturation step), 30 sec at 61 °C (annealing step) and 3¼ min at 68 °C (extension step). The PCR procedure was concluded by a10-minute incubation at 68 °C. The resulting 3.2-kilobase (kb) PCR fragment was purified with the aid of SureClean (Bioline) to remove dNTPs, primers and thermostable DNA polymerase molecules. Next, the PCR fragment was incubated with the restriction enzymes (REs) BamHI and XhoI (Fermentas), the 3.2-kb digestion product was extracted from agarose gel using JETSORB Gel Extraction Kit (GENOMED) and inserted downstream of the human cytomegalovirus immediate early gene (hCMV-IE) promoter, between the unique BamHI and XhoI of pcDNA3.1(+)/myc-His C (Invitrogen, catalog No.V80020). 

The construct pcDNA3.1-nls.lacZ(+) (Figure 1) was generated by insertion of the 3.2-kb nls.βgal-coding KpnI fragment of plasmid pMX1-nls.lacZ(+) (unpublished data) into the KpnI site of pcDNA3.1(+)/myc-His C (Invitrogen, catalog No.V80020). In addition to the lacZ expression unit, pcDNA3.1-nls.lacZ(+) contains a SV40 promoter-driven recombinant aphA1 gene that can be used to select stably transduced mammalian cells with the aid of the antibiotic G418 (also known as geneticin). The plasmid pcDNA3.1-nls.lacZ(-), which served as a negative control in the transfection experiment is identical to pcDNA3.1-nls.lacZ(+), except that the nls.βgal-coding KpnI fragment was inserted in the negative orientation with respect to the hCMV-IE promoter. Ligation reactions were performed with bacteriophage T4 DNA ligase from Fermentas and the ligation products were introduced into DH5αMCR cells (17). RE analyses of plasmid DNA extracted from cultures initiated with individual bacterial colonies were employed to identify bacteria containing the desired constructs. Large-scale preparations of pcDNA3.1-cyt.lacZ, pcDNA3.1-nls.lacZ(+) and pcDNA3.1-nls.lacZ(-) DNA were obtained using the JETSTAR Plasmid Midiprep Kit (GENOMED). The plasmid DNA was dissolved in sterile water at a final concentration of 1 µg/µl.

***Mammalian cell transfection***

Sixteen hours prior to transfection, 3.5×10^5^ HeLa cells were transferred to each well of a 6-well culture plate (SPL lifescience) and incubated overnight at 37 °C in a fully humidified atmosphere of 5% CO_2_ in air. The culture medium (2.5 ml per well) consisted of Dulbecco’s modified Eagle’s medium (DMEM; Euroclone) supplemented with 5% fetal bovine serum (FBS; Invitrogen), 100 µg/ml streptomycin (Cinnagen) and 100 U/ml penicillin (Cinnagen). Just before the start of the transfection procedure, 3 µl of pcDNA3.1-cyt.lacZ, pcDNA3.1-nls.lacZ(+) and pcDNA3.1-nls.lacZ(-) DNA were mixed with 102 µl of 150 mM NaCl and 27 µl of 1 µg/µl linear polyethylenimine (PEI; Mw ~25 kDa; Polysciences) was mixed with 288 µl of 150 mM NaCl. Next, 100 µl of each diluted plasmid sample was added to 100 µl of the diluted solution. After gentle vortexing for 10 sec, the samples were incubated for 15 min at room temperature to allow the formation of DNA/PEI complexes. In the meantime, the cell culture supernatant was replaced by 1 ml of fresh culture medium. Next the DNA/PEI complexes were added to the culture medium in a drop wise fashion. After incubation for 2 hr at 37 °C in an atmosphere of fully humidified air and 5% CO_2_, another ml of fresh culture medium was added. The next day, the cell culture supernatant was replaced by 2 ml of fresh culture medium.

***Cytochemical staining procedure***

At 48 hr post-transfection, the cell layers were washed for two times 5 min with phosphate-buffered saline (PBS) and fixed by incubation for 10 min at room temperature in PBS containing 0.25% gluteraldehyde. After two additional 5-min washes with PBS, the cells were incubated overnight at 37 °C in 5-bromo-4-chloro-3-indolyl-β-D-galactopyranoside (X-gal) solution (100 mM sodium phosphate [pH 7.0], 2 mM MgCl_2_, 5 mM K_3_Fe(CN)_6_, 5 mM K_4_Fe(CN)_6_, 1 mg/ml X-gal). The X-gal was added from a 40 mg/ml stock solution in 2.5% dimethylsulfoxide. Next, the cell layers were washed for two times 5 min with PBS containing 2 mM MgCl_2_. The staining result was evaluated using a Nikon TE-2000 inverse microscope equipped with Nikon camera 8400 for taking pictures of the cell layers.

**Figure 2.**
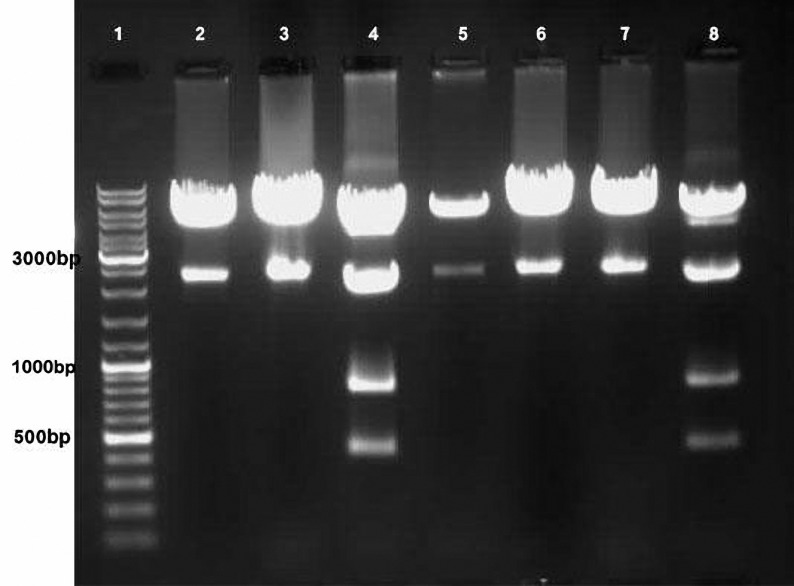
Agarose gel of MluI-digested plasmid DNA from bacteria transformed with the ligation mixture to generate pcDNA3.1-cyt.lacZ. Lane 1, Gene Ruler DNA Ladder Mix molecular weight marker (Fermentas). Lanes 2, 3, 5, 6 and 7, pcDNA3.1/myc-His C generated through self-ligation of the vector backbone. The minor DNA species represent denatured plasmid DNA. Lanes 4 and 8, pcDNA3.1-cyt.lacZ.

## Results

***Generation of nls-βgal- and cyt-βgal-coding mammalian expression plasmids***

The cyt.βgal-coding sequence was cloned into the BamHI and XhoI restriction enzyme site, the pcDNA3.1-cyt.lacZ yielded was inserted only in one correct direction. Agarose gel electrophoresis of MluI digests of plasmid DNA from bacteria transformed with the ligation mixture for generating pcDNA3.1-cyt.lacZ yielded 4 fragments 5295, 2089, 780 and 425 base pair (bp) which could be detected by the marker (Fermentas SM 1173), (Figure 2, Lanes 4 and 8). The other patters may have been arisen from the incomplete digestion of plasmid by either BamhI or XhoI. (Figure 2, Lanes 2, 3, 5, 6, 7). 

**Figure 3.**
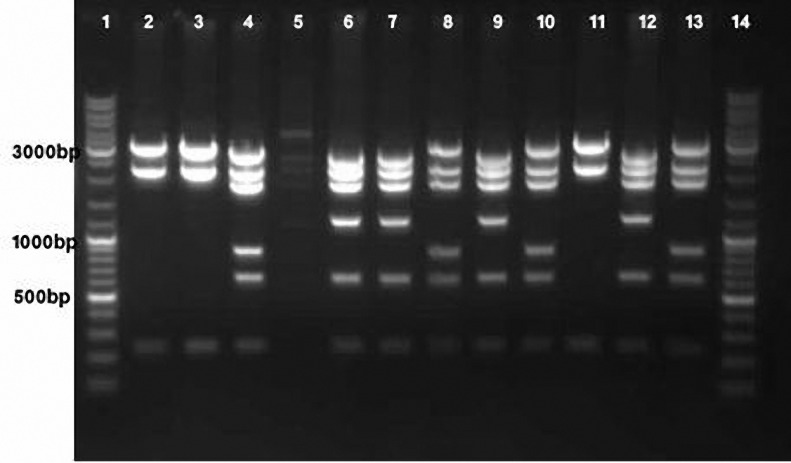
Agarose gel of HincII-digested plasmid DNA from bacteria transformed with the ligation mixture to generate pcDNA3.1-nls.lacZ(+). Lane 1, 14 Gene Ruler DNA Ladder Mix molecular weight markers (Fermentas). Lanes 2, 3, and 11, pcDNA3.1/myc-His C generated through self-ligation of the vector backbone. Lanes 4, 8, 10 and 13, pcDNA3.1-nls.lacZ(-). Lanes 6, 7, 9 and 12, with the proper orientation producing of pcDNA3.1-nls.lacZ(+), 2577, 2188, 1828, 1201, 624 and 234-bp fragments.

In contrast to the generation of pcDNA3.1-cyt.lacZ, which involved directional cloning into BamHI and XhoI RE recognition sites, pcDNA3.1-nls.lacZ(+) was generated through a nondirectional cloning

procedure utilizing a different restriction patterns were detected after HincII digestion of plasmid DNA from bacteria transformed with the ligation mixture for generating pcDNA3.1-nls.lacZ. These restriction patterns corresponded to self-ligated vector backbones (Figure 3, lanes 2, 3, 11) yielding fragments of 3067, 2188 and 234 base pairs (bp), pcDNA3.1/myc-His C derivatives containing the nls.βgal-coding sequence in the proper orientation producing 2577, 2188, 1828, 1201, 624 and 234-bp fragments (Figure 3, lanes 6, 7, 9, 12) and pcDNA3.1/myc-His C derivatives containing the nls.βgal-coding sequence in the wrong orientation yielding fragments of 2916, 2188, 1828, 862, 624 and 234-bp (Figure 3, lanes 4, 8, 10, 13).

***Transfection of HeLa cells with nls-βgal- and cyt-βgal-coding mammalian expression plasmids***

**Figure 4.**
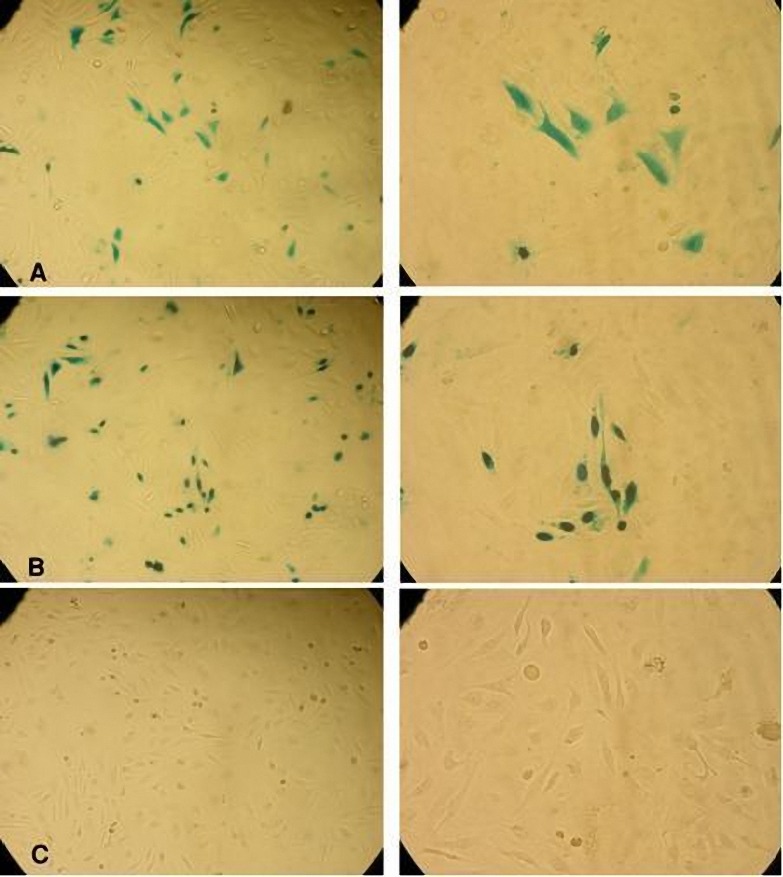
X-gal staining of HeLa cells transfected with pcDNA3.1-cyt.lacZ (panels A), pcDNA3.1-nls.lacZ(+) (panels B), and pcDNA3.1-nls.lacZ(-). (Panels C). Left column, ×100. Right column, ×200.

To test the functionality of pcDNA3.1-cyt.lacZ and pcDNA3.1-nls.lacZ(+), the plasmids were transfected into HeLa cells and subjected to an X-gal staining procedure at 48 hr after transfection. The negative control consisted of HeLa cells transfected with pcDNA3.1-nls.lacZ(-). As is evident fromFigure 4, panels A, after transfection with pcDNA3.1-cyt.lacZ, Hela cells displayed cytoplasmic βgal activity. In contrast, in pcDNA3.1-nls.lacZ(+)-transfected HeLa cells, βgal activity was largely confined to the cell nucleus (Figure 4, panels B). HeLa cells transfected with pcDNA3.1-nls.lacZ(-) did not show βgal activity (Figure 4, panels C).

## Discussion

In this study, we have demonstrated the utility of pcDNA3.1/myc-His C as nonviral vector for the expression of transgenes (i.e. lacZ) in mammalian cells (i.e. Hela cells). We also showed that in mammalian cells the *E. coli* βgal protein is localized in the cytoplasm but can be targeted to the cell nucleus by adding the nuclear localization signal of the SV40 large T protein (i.e. amino acids 127 through 147; 127KKKRKVEDPKDFPSELLSFLS147) to its N-terminus confirming the results obtained in several previous studies (12, 18,19). 

While comparing viral and nonviral vectors, the viral vector presents high efficiency and safety limitation; however, the nonviral vectors are safer but show relatively low level of gene delivery (20). Apart from the safety aspect, viral vectors transfer genetic material actively through the pores of nucleus. But nuclear entry of nonviral vectors still has significant limitation in plasmid transfection (21,22). In our study, the pcDNA3.1 containing nls sequences, which were inserted down stream of CMV promoter and up stream LacZ, caused the new generation pcDNA3.1-nls.LacZ(+) overcome the nuclear barrier by the activity of nls sequence which led the vector to pass from nuclear complex (Figure 1). We observed that the LacZ could be expressed in fraction of the HeLa cells. Although we cannot rule out the possibility that the sensitivity of our X-gal staining procedure was insufficient to detect all lacZ-expressing cells, these results most likely indicate that the nuclear delivery of the expression plasmids was suboptimal. 

Among the large collection of nonviral vectors, pcDNA3 and its derivatives stand out for their versatility, e.g. due to the presence in these plasmids of SV40 promoter-driven neomycin-, hygromycin- or zeocin-resistance genes. Recently, for pcDNA3.1(+) the repertoire of selectable markers has been expanded with blasticidin- and puromycin-resistance genes (23) further increasing the versatility of this nonviral vector system.

The pcDNA3.1 plasmid applied in our study is one of the enhanced plasmid, with hCMV IE promoters for high expression (Figure 1), therefore the HeLa cells were trancefected with the different cytoplasmic and nuclear variants. Recently, the enhanced pcDNA3.1 presents the high level expression in mammalian cells (24). Furthermore, the DNA vaccine is one of the new applications of pcDNA3.1 plasmids, which are relatively safe, efficient, and sustains the levels of antigen expression without immune responses compared with viral vectors (3,25). We showed that pcDNA3.1 has capacity for transgene expression and dominance over different barriers such as cytoplasmic membrane and nuclear envelope. Our vector presents the correct intracellular traffic and sustains its effective function of the recombinant *E. coli* βgal protein in cytoplasm and nucleus of HeLa cells (Figure 4). 

It has been stated that no more than 0.1-0.001% of cytoplasmically injected plasmid DNA gets transcribed (26). The use of physical transfection methods (e.g. electroporation) instead of chemical transfection agents like PEI may help to increase the percentage of transgene-expressing cells. Moreover, nonviral vectors could be endowed with sequences that allow tethering of the plasmid DNA to the nuclear matrix and thereby increase the number of transcription-competent templates (27). 

Finally, depending on the specific application, it may be necessary to replace the hCMV-IE promoter in pcDNA3 and its commercial derivatives by other promoters including those that can confer tissue-specific transgene expression (28). 

## Conclusions

The expression of the cytoplasmic and nuclear variant of LacZ in mammalian cells, demonstrate that the pcDNA3.1 is one of the versatility nonviral vectors for the experimental studies which provides a strong rationale for further exploration of nonviral vectors as gene delivery agents. It seems that for gene therapy it is necessary to optimize the designed vectors. 
